# Chilling alcohol on the computer: isothermal compressibility and the formation of hydrogen-bond clusters in liquid propan-1-ol

**DOI:** 10.1140/epje/s10189-023-00380-w

**Published:** 2023-11-29

**Authors:** Luis A. Baptista, Mauricio Sevilla, Manfred Wagner, Kurt Kremer, Robinson Cortes-Huerto

**Affiliations:** Max Planck Institute for Polymer Research, Ackermannweg 10, 55128 Mainz, Germany

## Abstract

**Abstract:**

Molecular dynamics simulations have been performed to compute the isothermal compressibility $\kappa _T$ of liquid propan-1-ol in the temperature range $200 \le T\le 300$ K. A change in behaviour, from normal (high *T*) to anomalous (low *T*), has been identified for $\kappa _T$ at $210<T<230$ K. The average number of hydrogen bonds (H–bond) per molecule turns to saturation in the same temperature interval, suggesting the formation of a relatively rigid network. Indeed, simulation results show a strong tendency to form H–bond clusters with distinct boundaries, with the average largest size and width of the size distribution growing upon decreasing temperature, in agreement with previous theoretical and experimental studies. These results also emphasise a connection between the behaviour of $\kappa _T$ and the formation of nanometric structures.

**Graphic Abstract:**

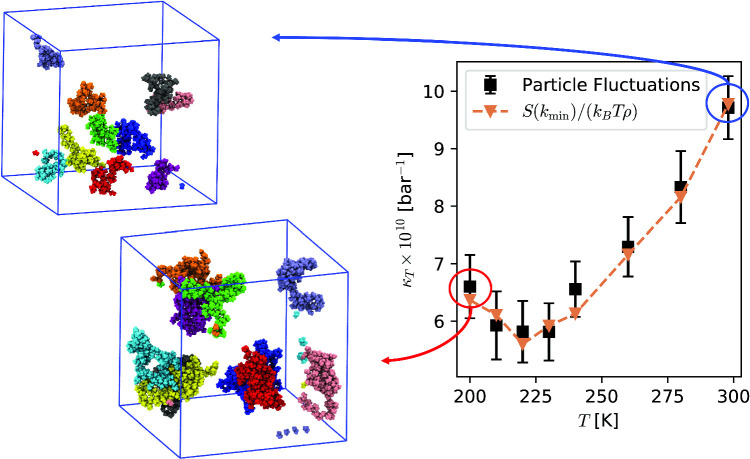

**Supplementary Information:**

The online version contains supplementary material available at 10.1140/epje/s10189-023-00380-w.

## Introduction

Hydrogen bonds (H–bonds) play a fundamental role in a wide range of phenomena in soft matter systems [1, 2], from the anomalous thermodynamic properties of water [3, 4], the solubility of (macro-)molecular synthetic and biological systems [5, 6] to the stabilisation and replication of DNA [7]. Collectively they are of significant relevance in supramolecular chemistry, where H–bonds are employed as one key concept to form (meta-)stable, functional polymer structures [8, 9]. The strength of H–bonds (4–8 $k_\mathrm{{B}}T$), with $k_\mathrm{{B}}$ being the Boltzmann constant and $T=300$ K, lies between the strong covalent bonds ($80~k_\textrm{B}T$ for a typical carbon-carbon bond) and the weak van der Waals interactions (less than $k_\textrm{B}T$). Consequently, individual H–bonds can rapidly form and break, with characteristic lifetime $\tau \sim 10^{-11}$ s [10]. As a result of that intermediate H–bond strength, directionality and rapid dynamics, many systems show a marked tendency to create networks that are highly temperature dependent [11, 12]. It is thus clear that, among others, the temperature dependence of H–bond interactions in molecular liquids is critical for achieving a fundamental understanding and developing engineered applications.

Indeed, thermoresponsive materials are now the focus of significant research efforts, given their encouraging range of applicability in various fields, including biology, energy, environment and materials science. A prominent example is given by smart, responsive polymers where even slight changes in the environment trigger substantial structural, stability and functional changes that can be tuned for advanced materials applications [13, 14] and pave the way for designing equivalent biocompatible materials [15]. Stimuli-responsive supramolecular assemblies also represent a case where a fine calibration of intermolecular interactions drives structure-bioactivity relationships [16]. Also, in the biological context, competing residue-osmolyte interactions are fundamental to understanding the denaturation of proteins [17]. Finally, thermoresponsive ionic liquid/water mixtures have been recently proposed as promising systems for applications in forward osmosis desalination, low-enthalpy thermal storage [18], and water harvesting from the atmosphere [19]. Common to all these phenomena and applications are their delicate corresponding temperature-dependant H–bond interactions. To better understand the combined effect of temperature and H–bond interactions, it is advantageous to investigate relatively simple systems containing the essential chemical characteristics and structure common to more complex organic fluids. Monohydroxy alcohols represent a prototypical system to investigate H–bond networks due to tunable hydroxyl (OH-) group density resulting from increasing or decreasing the alkyl chain length. In these systems, two molecules form an H–bond when the hydrogen in the OH group of one participates as the donor and the electronegative oxygen of the other as the acceptor. This indicates that a single OH group potentially forms up to three H–bonds, which results in molecular clusters exhibiting a wide variety of geometries [11, 12, 20, 21]. The dynamic properties of these H–bond structures have been extensively investigated in the literature. Dielectric response measurements in low-temperature propan-1-ol [12, 21–26], for example, reveal that in addition to the $\alpha$ relaxation process present in other liquids and related to structural rearrangements of the alkyl chain, there is a lower frequency relaxation process (Debye). Based on NMR measurements, the latter is believed to be induced by the H–bond network [20]. Its characteristic relaxation time $\tau _D$ varies strongly between $\tau _D \approx 10^{-10}s$ close to $T=330$ K and $\tau _d \approx 10^0\,s$ at about $T=125$ K. However, to the best of our knowledge, no computational studies in the literature address the thermodynamic effects, isothermal compressibility in particular, resulting from forming H–bond clusters in the system as a function of the temperature. A possible reason for this gap might be that, at low temperatures, one expects to see nanometric-size structures that imply considerably large system sizes and an appropriate description of finite-size effects. An approach to overcome these problems for computing bulk thermodynamic quantities from simulations of molecular liquids has been just recently developed by some of us [27–29]. This approach we now apply to this problem. Hence, in this work, we compute the isothermal compressibility $\kappa _T$ for samples of 20,000 propan-1-ol molecules in the liquid state in the temperature range $200<T<300$ K by molecular dynamics simulations. Following Ref. [20] $\tau _D$, the longest structural relaxation time in the system is expected to vary between $10^{-7}$ s ($T=200$ K) and about $3 \times 10^{-10}$ s ($T=300$ K). To account for that, all the samples were equilibrated for 100 ns. Moreover, our relatively large samples (simulation boxes of linear size $L\sim 13$ nm) minimise finite-size effects. Nevertheless, the spatial block analysis (SBA) method [27, 28], that takes into account explicit and implicit size effects, had to be used to accurately compute $\kappa _T$ in the thermodynamic limit. A change in the temperature dependence of $\kappa _T$, from normal (high T) to anomalous (low T) liquid behaviour, is apparent in the region $210<T<230$ K. Results from a hierarchical clustering method of H–bond networks in propan-1-ol reveal that the change in $\kappa _T$ with decreasing temperature is related to the formation of clusters of molecules connected via H–bonds.

## Computational details

**Fig. 1:**
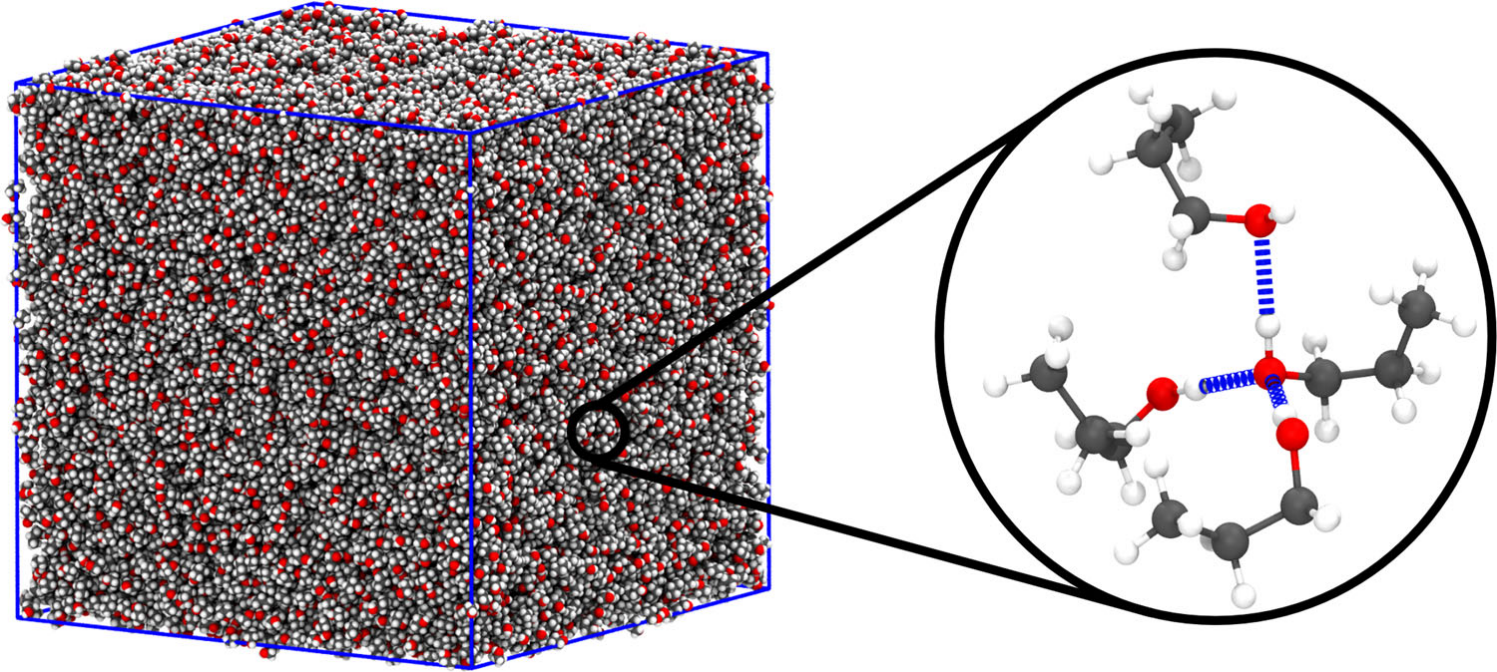
Simulation snapshot of the liquid propan-1-ol at $T=300$ K. A system containing 20,000 molecules occupies a volume with linear size $L \sim 13~\textrm{nm}$. The detail shows four propan-1-ol molecules connected via H–bonds, represented as dashed blue lines. The colour code for the atoms is as follows: H, white; C, grey and O, red

Simulations of liquid propan-1-ol in the NPT ensemble have been performed using the OPLS-AA force field [30], which includes bonded and non-bonded interactions. The former account for stretching, bending and torsion contributions from covalent bonds, whereas the latter include dispersion, via Lennard–Jones potentials, and electrostatic interactions. A typical simulation snapshot, indicating four propan-1-ol molecules connected via hydrogen bonds (H–bonds), is presented in Fig. 1. The sample occupies a cubic volume with applied periodic boundary conditions. Long-range electrostatic forces are treated via the smooth particle mesh Ewald algorithm [31]. Constant temperature and pressure conditions have been enforced using the velocity rescale thermostat [32] and the Parrinello-Rahman-Andersen barostat [33, 34], respectively. Simulations have been performed using the GROMACS 2019.6 [35] molecular dynamics simulation package with a time step of 2 fs. Samples of 20,000 propan-1-ol molecules have been quenched from $T=300$ K down to $T=240$ K in steps of $\Delta T = 20$ K, then from $T=240$ K to $T=200$ K in steps of $\Delta T = 10$ K. At each temperature, the system is equilibrated at NPT conditions for 100 ns, and statistics are accumulated, also at NPT conditions, for further 50 ns. The equilibration of the simulated samples has been controlled by monitoring the behaviour of the fluctuations of the number of particles for subdomains within the simulation box (See Right panel in Fig. 3). The system’s pressure is set to $P=1$ bar for all temperatures.

## Results and discussion

**Fig. 2:**
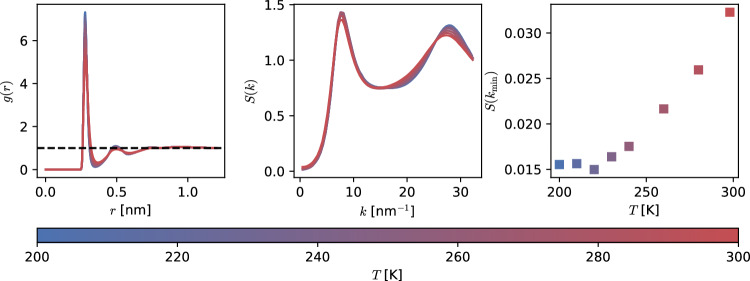
(Left panel) Temperature dependence of the radial distribution function *g*(*r*) and (Middle panel) structure factor *S*(*k*). (Right panel) $S(k_\mathrm{{min}})$ vs *T* with $k_\mathrm{{min}}\approx 0.48$ nm$^{-1}$ the minimum $k-$value in the reciprocal space discretisation

The investigation of structural properties starts with calculating the oxygen–oxygen radial distribution function, *g*(*r*), in the range of temperatures $200\le T \le 300$ K. As expected, the first and second peaks of *g*(*r*) weakly increase with decreasing temperature and the first minimum gets deeper with a marked shift towards smaller distances. See Fig. 2 (Left panel). Still, in the whole temperature range, these *g*(*r*) correspond to a liquid system, which is confirmed by the linear relation between average potential energy and temperature (result not shown).

**Fig. 3:**
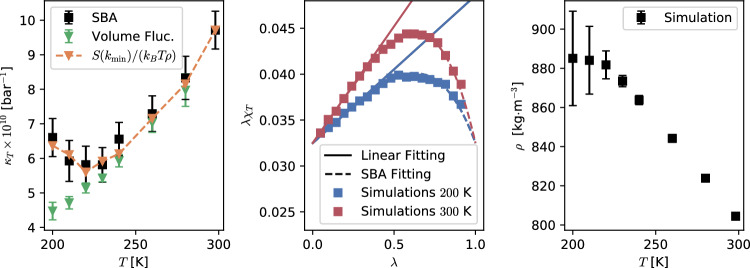
(Left panel) Isothermal compressibility as a function of temperature. The change from normal to anomalous behaviour is apparent in the interval $210< T < 230$ K when computing $\kappa _T$ with the SBA method, Eq. (6) (black symbols), in agreement with the behaviour exhibited by the $\lim _{k\rightarrow 0}S(k)$. By contrast, a direct calculation with Eq. (7) (Volume Fluctuations—green symbols) fails to capture the correct behaviour at low temperatures. In this case, small samples were used ($L\sim 5$ nm) to ensure convergence, with size comparable to the low-k portion of *S*(*k*) where the anomaly appears, i.e. $k<2$ nm$^{-1}$ ($L > 3$ nm). (Middle panel) Fluctuations of the number of particles $\chi _T(\lambda )$ as a function of the size of the subdomain $\lambda =(V/V_0)^{1/3}$ for two representative temperatures, $T=200~\textrm{and}~300~\textrm{K}$. The slope in the linear fitting, Eq. (6) with $\lambda \ll 1$, gives the bulk isothermal compressibility $\chi _T^{\infty }=\rho k_\textrm{B}T \kappa _{T}$. Using the obtained values for $\chi _T^{\infty }$ and $\alpha$ in Eq. (6), it is clear that the SBA method fits the whole simulation data set (SBA fitting). (Right panel) Average density as a function of temperature

Fluctuations in the long-range tail of the RDF encode information related to the thermodynamic properties of the liquid. This information becomes accessible from the simulation trajectory by computing the static structure factor, given by the expression:

1 $$\begin{aligned} S(k) = 1 + 4\pi \rho \int _{0}^{\infty }dr\, r^2\frac{\sin (kr)}{kr}(g(r)-1)\,, \end{aligned}$$

with *k* being the norm of a reciprocal-lattice vector, and $\rho$ the system’s average number density. To avoid numerical instabilities at the low-*k* region [36], typically the expression

2 $$\begin{aligned} S(\textbf{k}) = \left\langle \frac{1}{N}\sum _{i'=1}^{N}\sum _{j'=1}^{N}\exp {(-i\textbf{k}\cdot (\textbf{r}_{i'} - \textbf{r}_{j'} ))} \right\rangle \end{aligned}$$

is used, where the indices *i*, *j* refer to propan-1-ol oxygen atoms. The average runs over values of $k=|\textbf{k}|$ and the statistical ensemble. To compute *S*(*k*), we discretise the reciprocal space by considering vectors $\textbf{k}=2\pi (n_x, n_y, n_z)/L$ with *n* integer and a maximum value of $n_\mathrm{{max}}=70$. The results are presented in the middle panel of Fig. 2. The low height of the first peak in *S*(*k*) confirms that the system remains liquid in the temperature range considered [37, 38]. It can be seen that upon decreasing temperature, the first ($\sim 7$ nm$^{-1}$) and second ($\sim 27$ nm$^{-1}$) peaks become weakly more pronounced. More interestingly, in the range $k<2$ nm$^{-1}$ (Right panel Fig. 2), a change of behaviour becomes apparent with $\lim _{k\rightarrow 0}S(k)$ going from monotonically decreasing to a sudden increase starting at $T<220$ K. The overall shape of *S*(*k*), including the *anomalous* $k\rightarrow 0$ limit, is consistent with neutron diffraction measurements and related monte carlo simulations [12]. We recall that the limit $k\rightarrow 0$ of *S*(*k*) is related to the bulk isothermal compressibility $\kappa _T$ via the expression

3 $$\begin{aligned} \lim _{k\rightarrow 0} S(k) = \rho k_\textrm{B} T \kappa _T\,, \end{aligned}$$

with $k_\textrm{B}$ the Boltzmann constant. In practice, one evaluates $S(k_\mathrm{{min}})$ with $k_\mathrm{{min}}$ the minimum $k-$value in the reciprocal space discretisation, and the previous expression remains valid provided the system is homogeneous, i.e. $k_\mathrm{{min}}\ll 2\pi /\xi$ with $\xi$ being the system’s correlations length. Given the behaviour of *g*(*r*) and *S*(*k*) as discussed above, it is safe to assume that propan-1-ol in the temperature range $200~K \le T\le 300~k$ at $P=1$ bar is a homogeneous liquid. We present $S(k_\mathrm{{min}})/k_\mathrm{{B}}T\rho$ with $k_{\min }\approx 0.48$ nm$^{-1}$. Results can be seen in Fig. 3 (Left panel).

To compute $\kappa _T$, different finite-size effects present in the simulations prevent the use of the isothermal compressibility equation [37]

4 $$\begin{aligned} \chi _T^{\infty }{} & {} = \rho k_\mathrm{{B}}T \kappa _T = \frac{\Delta ^2(N)}{\langle N\rangle }\nonumber \\{} & {} = 1+4\pi \rho \int _{0}^{\infty }dr\, r^2 (g(r)-1)\,, \end{aligned}$$

with $\Delta ^2(N)=\langle N^2\rangle - \langle N\rangle ^2$. Instead, we use the spatial block analysis (SBA) method to extrapolate the bulk isothermal compressibility from finite-size computer simulations [27, 28, 39, 40]. Inside a simulation box of volume $V_0$, fluctuations of the number of molecules are computed for subvolumes *V*, using the expression

5 $$\begin{aligned} \chi _{T}(V;V_{0}) = \left. \frac{\Delta ^2(N)}{\langle N\rangle }\right| _{V;V_0}\,. \end{aligned}$$

The extrapolation of the isothermal compressibility in the thermodynamic limit $\kappa _T$ is obtained by systematically varying the size of the subdomain in the interval $0<V\le V_0$, and applying the formula [27, 28]

6 $$\begin{aligned} \lambda \chi _{T}(\lambda ) = \chi _{T}^{\infty }\left( 1 - \lambda ^{3}\right) \lambda + \frac{\rho \alpha }{V_{0}^{1/3}}\,, \end{aligned}$$

with $\lambda \equiv (V/V_{0})^{1/3}$, $V<V_{0}$, $\alpha$ being an intensive proportionality constant and $\chi _{T}(\lambda )\equiv \chi _{T}(V;V_{0})$. When $\lambda \ll 1$, $\lambda ^3\approx 0$ and $\chi _{T}^{\infty } = \rho k_\textrm{B}T \kappa _{T}$ can be extrapolated from a linear fit to the simulation data.

**Fig. 4:**
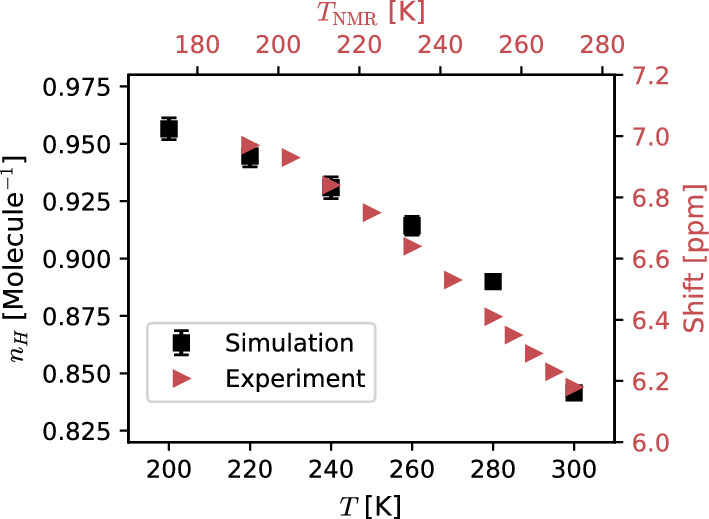
$n_{H}=\langle N_{H}\rangle /N$ and NMR chemical shift vs temperature. $\langle N_{H}\rangle$ is the average total number of H–bonds in the system and *N* is the total number of molecules. The value of $n_H$ is equivalent to counting all incoming H–bonds associated with O atoms and dividing it by twice the number of molecules. Simulation and experimental data show a rapid initial increase, followed by a turn to saturation at lower temperatures

An example of the use of the SBA method, Eq. (6), is given on the middle panel in Fig. 3 for two representative temperatures $T=200~\textrm{and}~300~\textrm{K}$. The overlap between the simulation data (symbols) and the SBA fitting (dashed lines) from Eq. (6) is, in our experience, a good indicator of the simulated samples being equilibrated [27]. The temperature dependence of $\kappa _T$ is presented on the left panel in Fig. 3 (black symbols). The agreement between the results from $S(k_\mathrm{{min}})/k_\textrm{B}T\rho$ and the SBA method further validate the use of Eq. (3). Reassuring the tendency observed with the $\lim _{k\rightarrow 0}S(k)$, $\kappa _T$ exhibits a deviation from the normal fluid behaviour visible in the interval $210<T<230$ K. The density as a function of temperature also indicates a change in the system’s thermodynamics upon cooling (See right panel in Fig. 3). The linear monotonic behaviour observed at high temperature saturates at temperatures below $T=230$ K. Interestingly, error bars increase systematically with decreasing temperature, signalling large fluctuations in the volume, compatible with an increase in $\kappa _T$. We note in passing that a common practice in the literature corresponds to computing $\kappa _T$ via the formula

7 $$\begin{aligned} \kappa _T=\frac{1}{k_BT\left<V_0\right>}\left( \left<V_0^2\right>-\left<V_0\right>^2\right) _\mathrm{{NPT}}\,, \end{aligned}$$

by evaluating volume fluctuations in simulations performed in the NPT ensemble. It has been reported that, to ensure convergence, this procedure requires significantly longer simulation times, especially at low temperatures [41]. For this reason, smaller systems (1000 propan-1-ol molecules, $L \sim 5$ nm) were simulated for $\sim 1~\mu \textrm{s}$ to maximise the statistical sample size. The results presented on the left panel in Fig. 3 (green symbols) indicate that using Eq. (7) gives markedly different results below $T=230$ K. We interpret this discrepancy by noting that the anomaly in *S*(*k*) becomes apparent for $k< 2$ nm$^{-1}$, whose characteristic size ($L > 3$ nm) is comparable to the system size ($L \sim 5$ nm). Moreover, it is not possible to resolve the relevant low-*k* portion of *S*(*k*) unambiguously with the $k_\textrm{min}\approx 1.26$ nm$^{-1}$ of the small samples (Data not shown). Finally, these results suggest that the SBA method, unlike volume fluctuations (Eq. (7)), provides an efficient and reliable calculation of bulk isothermal compressibilities. In the temperature range considered here, there is no apparent divergence of $\kappa _T$, thus there is no indication of a possible liquid-liquid phase transition [42, 43]. However, the formation of long-range nanometric domains has been widely proposed as the reason behind the anomaly in the low-k region of *S*(*k*) exhibited in various low-temperature liquids [44–46]. In a previous publication, some of us have found a similar anomaly in the structure factor in a supercooled binary mixture, identified with long-range concentration fluctuations [47]. However, for a single-component molecular system, the microscopic origin of the anomaly is not obvious. Therefore, in the following, this mechanism is discussed in more detail. It has been proposed that, upon supercooling, monohydroxy alcohols form transient *polymeric* chains, connected via H–bonds, responsible for the intense dielectric response at low frequencies (Debye peak) observed in these systems [11, 20]. A simple count of H–bonds as a function of temperature, as seen in Fig. 4, shows that the average number of H–bonds per molecule ($n_{H}$) increases monotonically upon decreasing temperature, tending to saturation at $T\sim 220$ K. This result emphasises the crucial role of H–bonds in dictating the resulting structural properties. Indeed, at low temperatures $n_{H}\rightarrow 1$, consistent with the transient polymer model discussed in the literature [11, 20]. NMR chemical shift as a function of temperature (see right y-axis in Fig. 4 and supporting information) indicates that hydrogen atoms in the hydroxyl group become more localised and increase their tendency to make directional bonds (with oxygen in a neighbouring –OH group) with decreasing temperature, in reasonable agreement with the simulation results. At this stage, it is still open whether the system forms nanostructures connected via H–bonds that increase their average size as temperature decreases.

**Fig. 5:**
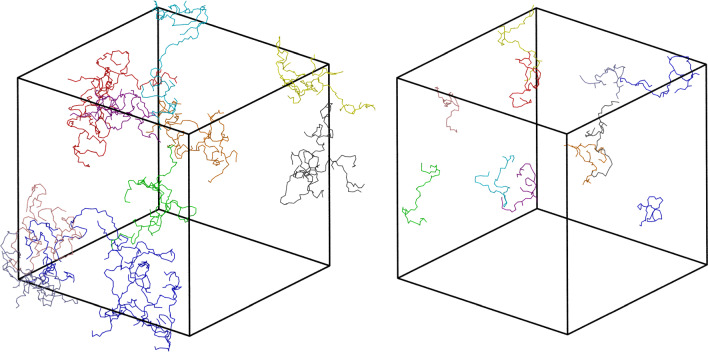
Ten largest H–bond clusters instantaneously present in a simulation snapshot at $T=200$ K (Left panel) and $T=300$ K (Right panel). The clusters are highlighted using the same colour for propan-1-ol molecules belonging to a geometrically defined H–bond network. The linear size of the simulation box is $L\sim 13$ nm

**Fig. 6:**
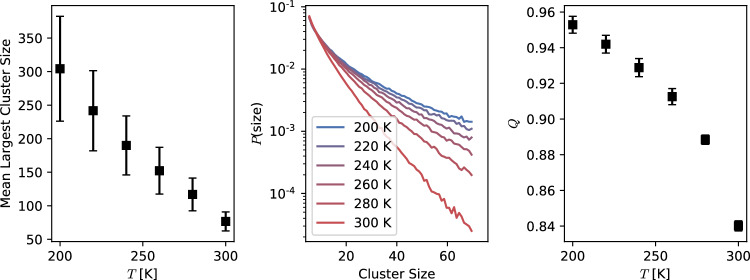
(Left) Temperature dependence of the mean largest cluster size. (Middle) Density distribution –Log scale– of H–bond clusters as a function of their size. (Right) Modularity *Q* of the H–bond network vs *T*

To investigate this possibility, propan-1-ol molecules were classified into disjoint clusters with elements connected via H–bonds and a statistical analysis was performed based on the clusters’ size distribution. We use a geometric definition of H–bonds, with donor–acceptor distance $\le 0.35$ nm and H–donor–acceptor angle $\le 30$ degrees. Donor and acceptor refer to O in propan-1-ol. The average number of H–bonds per molecule belonging to a cluster is $\sim 2$ at all temperatures. Snapshots of the ten largest clusters instantaneously present in a simulation are shown in Fig. 5 at $T=200$ K (Left panel) and $T=300$ K (Right panel), which resemble the polymer chains expected from experimental [20] and theoretical [11] studies. It is clear that at lower temperatures, clusters reach nanometric sizes ($\sim 300$ molecules), larger than the clusters visible at room temperature ($\sim 80$ molecules). These results are consistent with the transient-chain model [20], and agree well with statistical models and neutron diffraction experiments supporting the presence of clusters [11, 12, 23]. More interestingly, this cluster size dependence on temperature is systematic, with the mean largest-cluster size increasing upon decreasing temperature, as seen on the left panel in Fig. 6. The cluster size distribution shown in the middle panel in Fig. 6 agrees with this picture, with larger clusters appearing at lower temperatures.

The cluster population is analysed using a hierarchical clustering method [48–50]. In particular, we compute the modularity of the H–bond network. Namely, given a proposed network division into *communities* (clusters), it measures the quality of the division quantified in terms of a large number of edges (H–bonds) within communities and only a few between them. The degree $k_i$ of a vertex *i* in the system, i.e. the *i*th-oxygen atom, is defined as the number of incident H–bonds, that is

8 $$\begin{aligned} k_i = \sum _{j} A_{i j}\,, \end{aligned}$$

with the matrix

9 $$\begin{aligned} A_{ij} = {\left\{ \begin{array}{ll} 1~\text {if atoms}~(i,j)~\text {form a H--bond}\,,\\ 0~\text {otherwise}\,. \end{array}\right. } \end{aligned}$$

Finally, the modularity *Q* is defined as

10 $$\begin{aligned} Q = \frac{1}{2m}\sum _{ij}\left[ A_{ij} - \frac{k_i k_j}{2m} \right] \delta _{c_i,c_j}\,, \end{aligned}$$

with $m=\sum _{ij}A_{ij}/2$ a normalisation factor equal to the total number of edges, and $\delta _{c_i,c_j}$ a function with value one if the molecules (*i*, *j*) belong to the same cluster and zero otherwise. According to this definition, $Q=0$ corresponds to a homogeneous, randomised network. In contrast, values in the range $0.3<Q\le 1.0$ represent the tendency for the network to show a large community structure [48, 49], namely, the presence of clusters of highly-interconnected molecules with well-defined boundaries. The result of *Q* as a function of temperature is shown on the right panel in Fig. 6. It can be appreciated that even at high temperatures, the system has a marked tendency to form H–bond clusters with definite boundaries ($Q>0.8$), with substantially growing size upon decreasing temperature.

## Conclusions

In contrast to other organic liquids, low-temperature monohydroxy alcohols exhibit a simple dielectric behaviour identified with a Debye process, which results from the formation of stable H–bond structures. A vast body of theoretical and experimental literature has investigated these structures. However, to the best of our knowledge, a systematic investigation of temperature-dependent thermodynamic properties is somewhat lacking. Indeed, the formation of H–bond structures should be reflected by the system’s thermodynamics. To contribute to filling this gap, we compute the isothermal compressibility of propan-1-ol in the range of temperature $200\le T\le 300$ K. Our results show that the system transition from a normal (high *T*) to an anomalous liquid (low *T*) at $210< T < 230$ K, which can also be observed in the high-frequency region of the structure factor *S*(*k*). We find that the average number of H–bonds saturate starting approximately in the same temperature range, suggesting that the network increases its rigidity at this temperature. By classifying the propan-1-ol molecules into disjoint H–bond clusters, we investigate their size distribution using the hierarchical clustering method. These results reveal that clusters with well-defined boundaries form and increase their size upon decreasing temperature. The presence of these clusters is compatible with the behaviour of the isothermal compressibility $\kappa _T$. Indeed, $\kappa _T$ is related to the limit $S(k\rightarrow 0)$ that indicates the presence of large scattering domains with increasing sizes upon decreasing temperature. These scattering domains have been identified with H–bond polymeric chains, consistent with previous experimental and theoretical results [11, 20].

### Supplementary Information

Below is the link to the electronic supplementary material.

Supplementary file 1 (pdf 297 KB)

## Data Availability

Data will be made available upon reasonable request.
