# Supplementary material for: Chilling alcohol on the computer: isothermal compressibility and the formation of hydrogen-bond clusters in liquid propan-1-ol
Source: Eur Phys J E Soft Matter. 2023 Nov 29;46(11):117. doi: 10.1140/epje/s10189-023-00380-w (PMC10687148; doi:10.1140/epje/s10189-023-00380-w)
Supplement: Supplementary file 1 — (pdf 297 KB) [file 10189_2023_380_MOESM1_ESM.pdf]

Supplementary Material: "Chilling alcohol on the computer: isothermal compressibility and the nucleation of hydrogen-bond clusters in liquid propan-1-ol"

Luis A. Baptista,[1,*] Mauricio Sevilla,[1,*] Manfred Wagner,[1] Kurt Kremer,[1] and Robinson Cortes-Huerto[1,†]

[1] *Max Planck Institute for Polymer Research, Ackermannweg 10, 55128, Mainz, Germany*

This supplementary document provides additional supporting information for some of the results presented in the main draft.

S1. INTRODUCTION

Figure S1 shows the ^{1}H NMR spectra in the region of the hydrogen of the hydroxyl group of propan-1-ol as a function of temperature. It is possible to see two behaviours in the spectra: First, the signal is moving approximately 0.8 ppm towards lower field, from ∼6.2 to ∼7.0 ppm; second, the triplet signal is losing resolution. Upon decreasing temperature, hydrogen bonds become stronger. Hence, the proton moves away from the electronic density of the oxygen atom, causing the deshielding of the proton and the moving of the signal to a lower field. Moreover, this same constraint leads to a loss of the dynamics of the hydrogen atom. Therefore, the decrease in the temperature causes an increase in the local anisotropy around the hydrogen atom, leading to the broadening of the hydrogen signal.

Although on a minor scale, the decrease of excluded volume due to the decrease of the temperature also affects the other hydrogen atoms in the propan-1-ol. Figure S2 shows the ^{1}H NMR of propan-1-ol in the aliphatic carbons regions. It is possible to see the broadening of the signals due to the increase of anisotropy in the sample with cluster formation. Also, the small change (≈0.1 ppm) in the signal positions can be also attributed to the packing of the propan-1-ol molecules in the cluster formation with a decrease in temperature from 273.0 K to 193.0 K.

S2. EXPERIMENTAL SECTION

Propan-1-ol was purchased from Merck with at least 99.9 % (suitable for HPLC) of purity and was used without any further purification. NMR experiments were recorded in Bruker Avance III BR 500/51(500MHz) spectrometer. The experiments were carried out in a decreasing ramp of temperature.

*These authors contributed equally to this work.
†Electronic address: corteshu@mpip-mainz.mpg.de

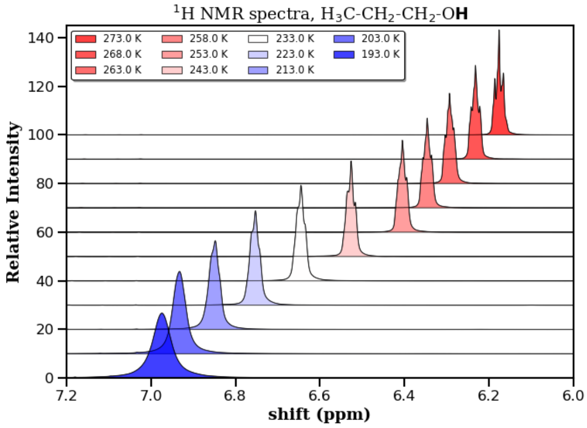

FIG. S1: ^{1}H NMR for the hydrogen in the hydroxyl group as a function of temperature. Upon decreasing temperature, the signal moves to low fields and the triplet signal, visible at high temperatures, turns into a Gaussian.

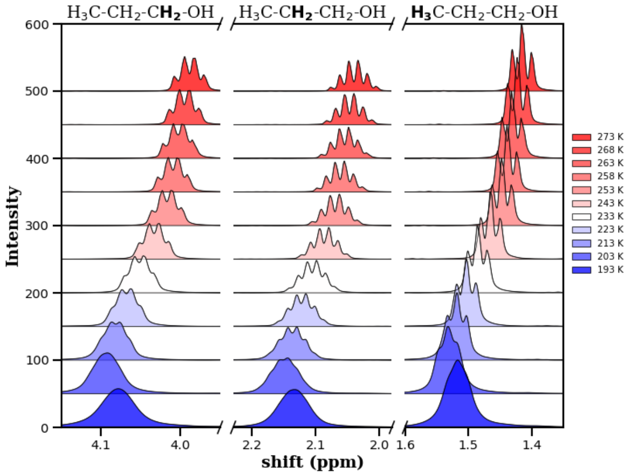

FIG. S2: A similar effect as observed in Fig. S1 is also present in the hydrogen atoms bonded to the aliphatic carbons.

S3. DESCRIPTION OF ^{1}H NMR SPECTRA

^{1}H NMR (500 MHz, tol-D_8, 273.0 K): δ 6.18 (t, J = 7.33, 1H), 3.99 (q, J = 6.18, 2H), 2.04 (m, J = 7.27, 2H), 1.42 (t, J = 7.33, 3H).
^{1}H NMR (500 MHz, tol-D_8, 268.0 K): δ 6.23 (t, J = 5.10, 1H), 4.00 (q, J = 6.17, 2H), 2.05 (m, J = 7.13, 2H), 1.42 (t, J = 7.49, 3H).

^{1}H NMR (500 MHz, tol-D_8, 263.0 K): δ 6.29 (t, J = 5.10, 1H), 4.00 (q, J = 6.15, 2H), 2.06 (m, J = 7.11, 2H), 1.43 (t, J = 7.48, 3H).
^{1}H NMR (500 MHz, tol-D_8, 258.0 K): δ 6.35 (t, J = 5.04, 1H), 4.01 (q, J = 6.16, 2H), 2.06 (m, J = 7.12, 2H), 1.44 (t, J = 7.48, 3H).
^{1}H NMR (500 MHz, tol-D_8, 253.0 K): δ 6.41 (t, J = 5.08, 1H), 4.02 (q, J = 6.15, 2H), 2.07 (m, J = 7.09, 2H), 1.45 (t, J = 7.50, 3H).
^{1}H NMR (500 MHz, tol-D_8, 243.0 K): δ 6.53 (t, J = 4.45, 1H), 4.03 (q, J = 6.02, 2H), 2.09 (m, J = 6.98, 2H), 1.47 (t, J = 7.48, 3H).
^{1}H NMR (500 MHz, tol-D_8, 233.0 K): δ 6.64 (s, 1H), 4.05 (q, J = 6.04, 2H), 2.11 (m, J = 7.18, 2H), 1.49 (t, J = 7.53, 3H).
^{1}H NMR (500 MHz, tol-D_8, 223.0 K): δ 6.75 (s, 1H), 4.07 (q, J = 6.01, 2H), 2.12 (m, J = 7.07, 2H), 1.50 (t, J = 7.50, 3H).
^{1}H NMR (500 MHz, tol-D_8, 213.0 K): δ 6.84, 4.09, 4.08, 2.16, 2.14, 2.13, 1.12, 1.52 (t, J = 7.36, 3H)
^{1}H NMR (500 MHz, tol-D_8, 203.0 K): δ 6.93, 4.09, 2.16, 2.14, 1.55, 1.53, 1.52.
^{1}H NMR (500 MHz, tol-D_8, 193.0 K): δ 6.97, 4.08, 2.13, 1.52.
